# Complete Genome Sequence of the Larvicidal Bacterium *Lysinibacillus sphaericus* Strain OT4b.25

**DOI:** 10.1128/genomeA.00257-16

**Published:** 2016-05-05

**Authors:** Andrés Rey, Laura Silva-Quintero, Jenny Dussán

**Affiliations:** Centro de Investigaciones Microbiologicas (CIMIC), Universidad de los Andes, Bogotá D.C., Colombia

## Abstract

*Lysinibacillus sphaericus* OT4b.25 is a native Colombian strain isolated from coleopteran larvae in an oak forest near Bogotá D.C.; this strain has shown high levels of pathogenic activity against *Culex quinquefasciatus* larvae in laboratory assays compared to that of other members of the same species. Using Pacific Biosciences sequencing technology, we propose a chromosomal contig of 4,665,775 bp that, according to comparative analysis, is highly similar to that of reference strain *L. sphaericus* C3-41.

## GENOME ANNOUNCEMENT

*Lysinibacillus sphaericus* is an aerobic, Gram positive, spore-forming bacterium, widely used in biological control of vector-borne diseases, like dengue, chikungunya, and Zika, all of which are considered major public health issues (1), due to its highly lethal larvicidal action (2, 3). Some strains are reported to be highly toxic against several mosquito species, such as *Culex* sp., *Anopheles* sp., and *Aedes* species (4). The fact that *L. sphaericus* pathogenic effects are limited against insects, such as *Culex* sp. and *Aedes* sp., is of major interest in the biological control of vector-borne diseases. Strain *L. sphaericus* OT4b.25 was isolated from beetle larvae from an oak forest near Bogotá D.C., Colombia, with many attributed biological activities, such as tolerance to toxic metals, like arsenic, chromium, lead, and cobalt (5, 6), and a high level of larvicidal activity against *Culex quinquefasciatus* larvae. Genomic DNA was extracted and purified using the GeneJET genomic DNA purification kit (catalog no. K0721; Thermo Scientific), extending incubation time with lysis buffer to 1 hour and doubling the recommended lysozyme concentration. Genomic DNA samples were prepared for a Pacific Biosciences RSII small-insert circular consensus sequence library and then were sent to Génome Québec (Montreal, Canada); genomic assembly was done according to the HGAP workflow (7). This resulted in an assembly with a total 4,841,658 bp, G+C content of 37.15%, and an estimated coverage of 97.0×. The assembly yielded two contigs, the first one of 4,665,575 bp and the second one of 176,083 bp. The genomic sequences were annotated using the prokaryotic annotation server RAST (8), Blast2Go (9), and BLAST. The possible orthologs present in the genomic contig were identified based on the COG database and classified accordingly (10). Then, the sequences were deposited at the Short Read Archive (SRA) and GenBank. Annotation revealed a total of 4,627 open reading frames with 147 RNA genes, with 32 of them being tRNA genes, and 2,470 (53.38%) of the initial 4,627 genes were assigned within the 25 COG functional categories. The genome of *L. sphaericus* OT4b.25 shows a wide repertoire of protein-coding sequences in terms of mosquitocidal toxins and genes important to larvicidal activity, containing coding sequences for toxins *binA* and *binB* coding for both a putative hemolysin and hemolysin D; interestingly, both coding sequences showed no significant similarity compared to those previously reported with the sphaericolysin B354 of *L. sphaericus* OT4b.31 (11) but showing similarity with hemolysin genes reported in *L. sphaericus* C3-41. We can confirm the presence of coding sequences for a chitin deacetylase and two chitin-binding proteins reported in *L. sphaericus* CBAM5. The genome contains 16 coding sequences for S-layer and S-layer-like proteins; these proteins have shown direct involvement in larvicidal activity (12). All of these are genes that are considered virulence factors in the pathogenic process of *L. sphaericus* against insect larvae, hemolysins, and chitin degradation mechanisms present a novel approach towards understanding the larvicidal activity of *L. sphaericus*.

### Nucleotide sequence accession numbers.

The GenBank accession numbers for contigs 1 and 2 are CP014643 and CP014644, respectively.
